# Potential biomarker for diagnosis and therapy of sepsis: Lactylation

**DOI:** 10.1002/iid3.1042

**Published:** 2023-10-13

**Authors:** ZeXian Sun, Yu Song, Jie Li, Yize Li, YongHao Yu, Xin Wang

**Affiliations:** ^1^ Department of Anesthesiology Tianjin Medical University General Hospital Tianjin China; ^2^ Anaesthesiology, The Graduate School Tianjin Medical University Tianjin China

**Keywords:** epigenetic modification, glycolysis, lactate, lactylation, sepsis

## Abstract

**Introduction:**

As a disease that has plagued human health for decades, sepsis has so far had no specific diagnostic or therapeutic indicators. The discovery of lactylation modifications not only uncovered the deep‐rooted causes of changing between lactate level and pathophysiology and immunology of sepsis, but also reaffirmed the inevitable link between metabolic reprogramming and epigenetic reprogramming in sepsis. Lactylation modification became a potential marker for diagnosis and guiding the treatment of sepsis.

**Aim:**

In this paper, we will summarize the discovery and regulation of lactylation modifications, discuss the study of lactylation modifications in sepsis, and evaluate their possibility and potential as diagnostic and therapeutic indicators of sepsis.

**Conclusion:**

Lactylation modification is directly regulated by glycolysis and lactate, and inhibition of glycolytic pathway‐related enzymes can regulate the level of lactylation modification, and more importantly, lactylation modification can act on these enzymes to regulate their functions and feedback regulate the level of glycolysis, this finding provides more ideas for clinical treatment of sepsis. We use “epigenetic modification”, “glycolysis”, “lactate”, “lactylaiton” and “sepsis” as keywords and search the relevant literature through Pubmed and Web of science up to 2023.

## Graphical abstract

Lactylation is a biomarker regulated by glycolysis in sepsis. Sepsis promotes the process of glycolysis, causing histones and nonhistones to be modified by lactylation. Lactylation can indicate not only inflammation but also immune status. Modulation of the glycolysis can inhibit excessive lactylation and reduce mortality of septic mice.

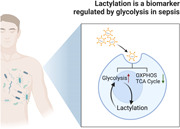

## 1 INTRODUCTION

Globally, there are more than 48.9 million new cases of sepsis each year and about 11 million deaths, accounting for 20% of global deaths.^1^ After decades of research, the mechanism of sepsis and its diagnosis and treatment are still difficult problems, and most of them are based on supportive therapies such as hemodynamic stabilization, antibiotic therapy, and maintenance of organ function, with no specific diagnostic and therapeutic measures yet.^2^, ^3^ The altered energy metabolism that accompanies sepsis patients has gradually become a focus of attention for researchers in recent years. Transcriptional and metabolic analyses of sepsis patients have shown that the transition from oxidative phosphorylation to aerobic glycolysis is an important component of the initial activation of host defenses.^4^ The glycolytic product, lactate, is an important indicator of the severity and mortality of sepsis.^5^, ^6^, ^7^ Both increased lactate production (e.g., hypoxia, increased aerobic glycolysis, and abnormal aerobic metabolism due to impaired mitochondrial function) and decreased lactate clearance lead to lactate accumulation in sepsis patients.^8^ Early reduction of serum lactate levels is one of the goals of intensive sepsis treatment,^9^, ^10^ but the reduction of serum lactate levels in the later stages of sepsis does not significantly improve the prognosis.^11^

The idea that lactate is the “ugly duckling” of energy metabolism has been overturned, and there is growing evidence that lactate is not a temporary metabolite in the absence of oxygen, but a fundamental member of energy conversion through blood circulation.^8^ It can perform anti‐inflammatory, immunomodulatory, and tissue repair functions through intracellular shuttling and intercellular shuttling.^12^ However, how lactate plays a regulatory role in the immune imbalance of sepsis remains a mystery.

In 2019, Prof. Yingmin Zhao's team discovered a novel acylation modification, lactylation modification, and demonstrated that lactate can directly promote histone lactylation and regulate protein gene expression.^13^ This discovery combines glycolysis and epigenetics, and also opens up new ideas for the study of sepsis. In this paper, we will summarize the discovery and regulation of lactylation and the current research in sepsis and analyze the prospects of its application in the diagnosis and treatment of sepsis.

## 2 ALTERATIONS AND ROLE OF LACTATE AND METABOLIC REMODELING IN SEPSIS

International sepsis guidelines recommend early monitoring of patients' blood lactate levels and timely interventions to reduce patients' serum lactate levels (a lactate concentration >2 mmol/L indicates a potential tissue perfusion deficit).^7^, ^14^, ^15^ Elevated blood lactate levels in sepsis patients are the result of a combination of factors, but the massive mobilization of immune cells involved in the inflammatory response to sepsis is the main cause of lactate production and accumulation.^16^

During the hyperinflammatory phase of sepsis, apoptosis and 10%–20% persistent lymphocyte death lead to lymphocytopenia, so the body needs to differentiate new lymphocytes rapidly and in large numbers to clear the pathogens from the body.^17^ This process, which uses efficient phagocytosis to destroy antigens and mediates an intense inflammatory response, is similar to the “Warburg effect” present in tumor cells.^18^, ^19^ Although glucose metabolism under aerobic conditions can produce more ATP than under anaerobic conditions,^20^ in some special cases, such as sepsis, immune cells tend to obtain energy rapidly through glycolysis as a way to help their rapid proliferation and differentiation to ensure a high‐intensity inflammatory response, to clear pathogens as early as possible.^21^, ^22^ During this process, lactate is produced in large quantities and inevitably accumulates in the body.^22^ For a long time, the idea that lactate is a metabolic waste product was dominant, but with the advancement of science, this stereotype has been disproved.^8^

The new view that aerobic glycolysis in the body continues to produce lactate has replaced the old view that lactate is only produced and accumulated under anaerobic conditions. Nowadays, lactate is no longer a metabolic waste product, but is considered as an important source of energy and a precursor and signaling molecule for gluconeogenesis.^23^

## 3 THE FINDINGS OF LACTYLATION

In an attempt to digest the core histones of Michigan Cancer Foundation‐7 for high‐performance liquid chromatography (HPLC)‐tandem mass spectrometric (MS/MS) analysis, Prof. Yingming Zhao's team detected the same mass transfer (Δ*m* = 72.021 Da) on the lysine residues in the three proteolytic peptides as that induced by the addition of lactyl groups to the ε‐amino groups of the lysine residues.^13^

They subsequently demonstrated the presence of histone lactylation modifications using four orthogonal approaches. In addition, they developed a pan‐antilactylation antibody and reconfirmed the presence of histone lactylation modification by western blot and showed that exogenous l‐lactate increased the level of histone lactylation in a dose‐dependent manner. Metabolic labeling experiments with the isotope sodium l‐lactate (^13^C_3_) suggested that lactylation of lysine can be derived from lactate.^13^ Together, these experimental results demonstrate that histone lactylation is an in vivo protein posttranslational modification (PTM) derived from lactate.

## 4 THE REGULATION OF LACTYLATION

U‐^13^C_6_ glucose labeling experiments showed that the endogenous metabolic pathway by which cells obtain energy through the catabolism of glucose is a key pathway for lactylation.^13^ When 2‐deoxy‐d‐glucose (2‐DG), a nonmetabolizable glucose analog, was used instead of glucose, both lactate and lactylation levels were reduced.^13^, ^24^

**Figure 1:**
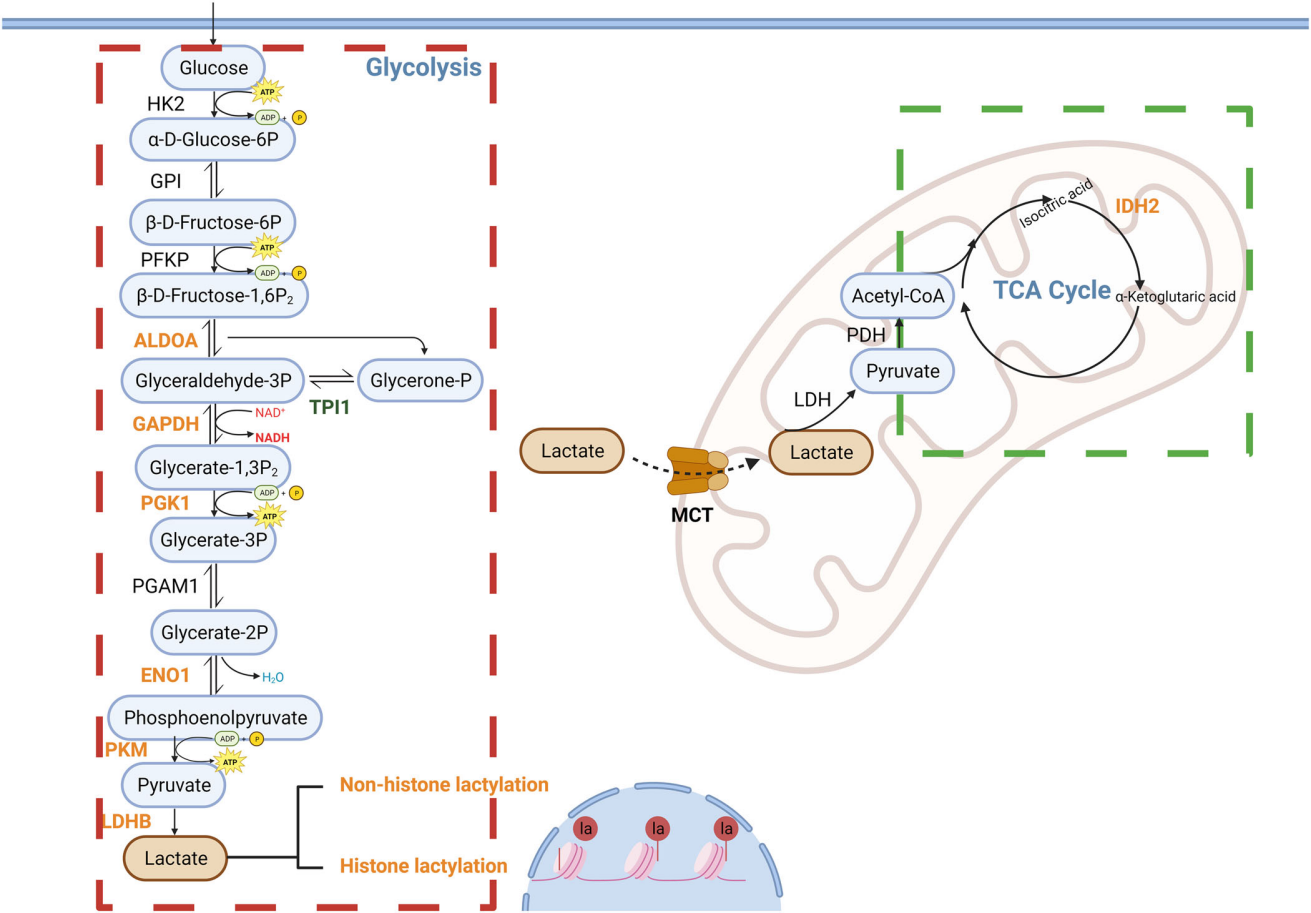
Lactate production by the glycolytic pathway regulates the modification of histone and nonhistone lactylation under sepsis conditions. Red box indicates that glycolysis tends to be upregulated, green box indicates that the tricarboxylic acid cycle tend to be downregulated, and this alteration in metabolic homeostasis is commonly found in tissues and cells following sepsis or lipopolysaccharide (LPS) induction. The orange‐labeled enzymes were all found to be modifiable by lactylation, and although the function of these enzymes after modification remain undefined, it is certain that the glycolysis‐related enzymes that have a higher propensity to be modified by lactylation. In addition to mediating lactylation modification, lactate is also transported into the mitochondria by the monocarboxylate carrier (MCT),^23^ where it is converted to pyruvate by lactate dehydrogenase to participate in the tricarboxylic acid cycle.^25^

The production of lactate depends on the balance between glycolysis and mitochondrial respiration. Sodium dichloroacetate (DCA) and sodium oxamate inhibit pyruvate dehydrogenase (PDH) and lactate dehydrogenase (LDH) activities of glycolysis, respectively, which inhibit intracellular lactate production and reduce histone lactylation levels.^13^ In contrast, inhibition of the oxidative respiratory chain in mitochondria by rotenone promotes glycolysis and increases intracellular lactate and histone lactylation levels.^13^ The increase of exogenous lactate could also increase the lactylation level of histones.^13^ It was demonstrated that glycolysis level was a determinant of lactylation modification and lactate was directly responsible for promoting lactylation modification (Figure 1).

## 5 HISTONE LACTYLATION

**Figure 2:**
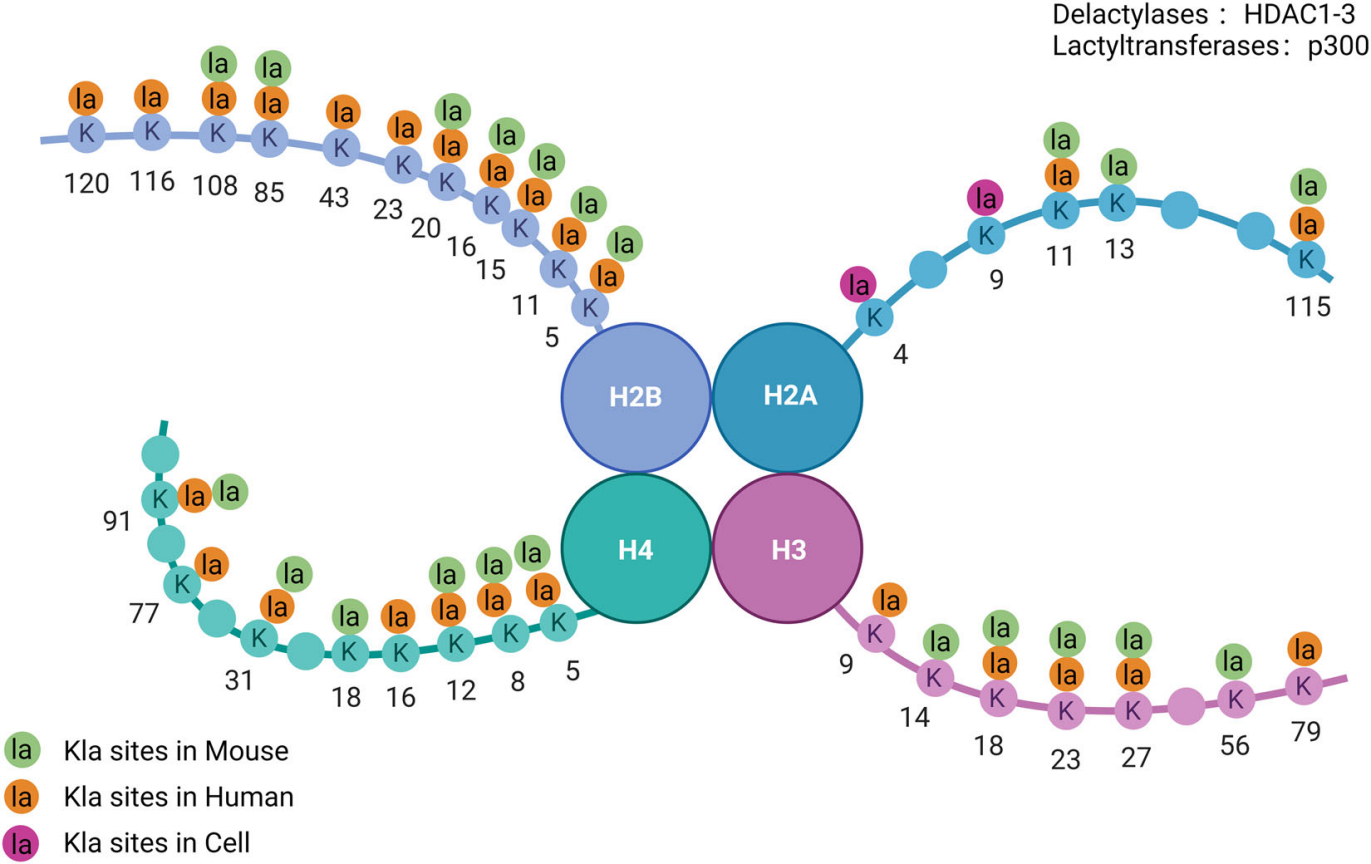
Sites of histone lactylation. Sites of lactylation identified on four core histones, H3, H4, H2A, and H2B. Numbers represent the different sequences of lysines in histones. Green circles represent humans, orange circles represent mice and purple circles represent cells.

In eukaryotic cells, the basic unit of chromosomes is the nucleosome. Two H2A–H2B dimers and one H3–H4 tetramer form an octamer, and 200 bp of DNA is wrapped around its surface to form a complete nucleosome.^26^ H1 connects adjacent nucleosomes in series to form a chromosome containing all the genetic information of an individual.^27^ PTM of histones is an important component of epigenetics, where different acyl groups can be attached to amino acid residues at the amino and carboxyl termini of histones (H1, H2A, H2B, H3, H4).^28^ This covalent modification regulates the tightness of histone and DNA binding, regulates the openness of gene loci, affects gene expression, and leads to transcriptional activation or gene silencing.^29^ The sites of histone lactylation are summarized in Figure 2.

In the earliest studies, histone H3 lysine 18 lactylation (H3K18la) was found to be involved in the expression of homeostatic genes for damage repair, such as Arg‐1.^13^ This suggests some role for H3K18la in inflammation‐related diseases. In a clinical study on sepsis, investigators collected peripheral blood mononuclear cells from healthy volunteers and 35 critically ill patients (septic and nonseptic) and examined them using multiple experimental techniques and found that^30^: (1) H3K18la was commonly expressed in peripheral blood mononuclear cells from healthy and critically ill individuals, but H3K18la was significantly elevated in patients in shock, demonstrating that H3K18la is suggestive for extreme critical illness; (2) H3K18la levels were positively correlated with procalcitonin (PCT) and negatively correlated with monocyte percentage, making it clear that high H3K18la expression is associated with infection, and high levels of H3K18la may be expected to be a new indicator of infection, but more clinically relevant data are still needed to prove this; (3) H3K18la was strongly and positively correlated with interleukin (IL)‐6 and IL‐10, and with Arg‐1 mRNA expression was also positively correlated.

In mouse experiments, methylsulfonylmethane could induce macrophage polarization toward wound‐healing type by upregulating H3K18la levels, resulting in decreased plasma levels of pro‐inflammatory cytokines and increased levels of anti‐inflammatory cytokines in septic mice infected with methicillin‐resistant *Staphylococcus aureus*, thereby alleviating the excessive inflammatory response and improving mouse survival.^31^ Because lactylation modification is a consequence rather than a cause, these findings clarify the role of H3K18la in the recognition of sepsis and its potential as a diagnostic indicator of septic shock or a prognostic indicator of sepsis, which is expected to assist in the clinical assessment of the immune status of the patient's organism and guide clinical treatment.

## 6 NONHISTONE LACTYLATION

Although lactylation modification was first identified on histones, it is widely found in the nucleus and cytoplasm, mitochondria, endoplasmic reticulum, and cytoskeleton. Hui Ye's team found that at *m*/z 156.103, a strong CycIm ion was generated after the loss of NH_3_, which inherits the three‐carbon unit of lactate and then produces a mass shift at *m*/*z* 159.1125, and this mass shift difference is where the lactylated peptide exhibits a mass shift (Δ*m* = 3.0101 Da).^32^ This demonstrates that CycIm ions can track proteins and sites modified by lactylation with relative accuracy, leading to the finding that lactylation modifications are prevalent in human and mouse cells and exhibit high enrichment in glycolysis‐related enzymes (Figure 1).

The lactylation of the K147 site of aldolase A (ALDOA) was the most frequent, and when the K147 site was modified by lactylation, the activity of ALDOA decreased, which inhibited glycolysis and reduced lactate production, forming a negative feedback regulation of glycolysis.^32^ Lysine residues of enolase 1 (ENO1) that could be modified by lactylation accounted for the highest percentage, and the lactylation modification of K343 and K326 regulated the thermal stability of ENO1.^32^ Numerous studies have demonstrated that pyruvate kinase M2 (PKM2) plays a key role in regulating the Warburg effect. Lactylation of the K62 site of PKM2 inhibits glycolysis and induces a shift in macrophages toward a repair phenotype, leading to upregulation of Arg‐1 expression and ultimately promoting wound healing in wound model mice.^33^ It is evident that glycolysis‐related enzymes can regulate lactylation modifications through lactate levels, and it was also found that these enzymes themselves can be modified by lactylation and affect glycolysis. Although this feedback regulatory loop still needs more exploration and refinement, the present findings suggest that the link between metabolic reprogramming and epigenetic alterations is complex and tight.

In addition to glycolysis, enzymes in the tricarboxylic acid cycle, carbohydrate, amino acid, fatty acid, and nucleotide metabolic pathways are modified by lactylation. The association between lactylaiton of metabolism‐related enzymes and hepatitis B virus‐associated hepatocellular carcinoma (HCC) has been confirmed by global lactase profiling and the finding that lactylation of K28 inhibits the function of adenylate kinase 2 (AK2) and promotes the proliferation and metastasis of HCC cells.^34^ This also suggests that more inflammation‐related diseases, such as sepsis, can find more specific enzymes and loci through lactate lyase profiling secretion to investigate their regulatory or indicative role in disease development.

High mobility group protein 1 (HMGB1) levels are predictive of survival in advanced sepsis.^35^, ^36^ Lactate not only leads to the accumulation of HMGB1 in macrophages, but also inhibits the deacetylase silencing information regulator 1 (SIRT1) activity and recruits the acetylase CBP/p300, promoting lactylation modification and acetylation modification of HMGB1.^37^, ^38^ The accumulated HMGB1 is released into the blood via exosomes, and ultimately septic mice have increased mortality.^37^

The above findings confirm the inevitable association of histone and nonhistone lactylation modifications with diseases such as infection‐associated diseases and sepsis, and also indicate its potential as a reference indicator for diagnosis of sepsis diseases, determination of disease severity, judgment of prognosis, and guidance of treatment.

## 7 RELATIONSHIP BETWEEN LACTYLATION AND ACETYLATION

Studies have shown that the relationship between lactylation and acetylation is relatively close, and the interrelationship between them is also a difficult and hot topic.

Lactate and acetyl coenzyme A (acetyl‐CoA) are both end products of pyruvate in glycolysis. Lactate is produced in the cytoplasm and mediates histone and nonhistone lactylation modifications,^13^ acetyl‐CoA is produced in the mitochondria and can participate in both acetylation and the subsequent tricarboxylic acid cycle.^39^ Pyruvate can efficiently shuttle between the cytoplasm and mitochondria.^40^ Pyruvate is perhaps the key molecule in maintaining lactylation and acetylation homeostasis.^41^ In sepsis, complex molecular alterations disrupt the dynamic homeostasis that exists between acetylation and lactylation modifications.

CBP/p300, a histone acetyltransferase and transcriptional cofactor,^42^ was the first enzyme shown to mediate lactylation modification.^13^ Histone deacetylase 1–3 (HDAC1–3) and silencing SIRT1–3 are the eraser of lysine acetylation regulation and have been shown to catalyze lysine delactylation, but no stable pattern of action and outcome has been found about SIRT1–3, and further studies are needed.^43^ Because both lactyl and acetyl groups are derived from the glucose metabolic pathway, and the related enzymes known to regulate lactylation modifications are also related to acetylation regulation, some scholars believe that histone lactylation and acetylation are in a dynamic equilibrium,^41^ but the way this equilibrium is constructed is still unknown, because histone lactylation and acetylation modifications exhibit different tendencies to change.^13^

## 8 POTENTIAL RELEVANT AGONISTS OR INHIBITORS OF LACTYLATION REGULATION IN THE TREATMENT OF SEPSIS

The level of lactylation modification is directly regulated by the level of lactate, which in turn determines the production of lactate, so the rate‐limiting enzyme that regulates glycolysis is one of the keys to interfere with lactylation modification (Table 1).

**Table 1:** Agents targeting deregulated glycolysis for the treatment of sepsis preclinical studies.

Compounds	Mechanism of action	Functions in sepsis	References
2‐DG	An analog of glucose to inhibit HK2 activity	Improves in histopathology, pulmonary vascular permeability, inflammatory cell infiltration, oxidative stress damage, and overexpression of pro‐inflammatory factors of lung; reverses the promotion of autophagy in kidney.	[24, 44]
Lonidamine	Inhibits HK2	Has anti‐inflammatory effects via suppressing the release of pro‐inflammatory cytokines in sepsis.	[45, 46]
Sodium oxamate	Inhibits LDHA	Lower the level of HMGB1, lactate, and lactylation, improve the survival outcome of septic mice.	[37]
Chrysin	Inhibits HK2	Raises HO‐1 to reduce oxidative stress and inflammation, reverses functional damage in multiple organs such as heart, lung, liver, and kidney, and optimizes histopathological scores, improves sepsis survival by LPS.	[47, 48]
Genistein	Directly downregulates HIF‐1α, therefore, inactivating GLUT1 and HK2	Improves cognition impairment in septic rats via decreasing inflammatory responses and oxidative stress, and activation of the Nrf2 pathway; Protects LPS‐induced lung vascular endothelial cells apoptosis by the Myd88/NF‐κB/BCL‐2 signaling pathway.	[49, 50]
Emodin	Decreases glycolytic enzymes expression (HK2, PKM2, and LDHA)	Protect intestinal mucosa through the VDR/NRF2/HO‐1 signaling pathway, improve remote liver and lung injury caused by CLP; Improves LPS‐induced myocardial injury and cardiac dysfunction by reducing NLRP3 and GSDMD expression.	[51, 52]
Shikonin	Inhibits PKM2	Improve sepsis‐induced lung injury by upregulated miRNA‐140‐5p expression; Improve sepsis‐induced acute kidney injury in rats, and attenuate the LPS‐induced kidney tubular epithelial cells apoptosis via modulating NOX4/PTEN/AKT pathway; Inhibit SIRT1‐dependent activation of NLRP3 inflammasomes to ameliorate LPS‐induced cardiac dysfunction.	[53, 54, 55]
Cel	Inhibits PKM2 and LDHA activity	Inhibits the level of pro‐inflammatory cytokines to improve survival in septic mice.	[56]
4‐OI	Inhibits GAPDH activity	Prevents macrophage activation and reduces serum levels of IL‐1β, IL‐6, and lactate in septic mice.	[57]
Tiliroside	Decreases glycolytic enzymes expression (GLUT1, ENO1, PKM, PDK1, LDHA, PGAM, PFK, GAPDH)	Modulates macrophages function and has the anti‐inflammatory effects.	[58]

Abbreviations: 2‐DG, 2‐deoxy‐d‐glucose; 4‐OI, 4‐Octyl itaconatek; BCL‐2, B‐cell lymphoma‐2; Cel, celastrol; CLP, cecum ligation and puncture; GSDMD, gasdermin D; HIF‐1α, hypoxia inducible factor‐1 alpha; HK2, hexokinase 2; HO‐1, heme oxygenase‐1; IL, interleukin; LDHA, lactate dehydrogenase A; LPS, lipopolysaccharide; NF‐κB, nuclear factor kappa‐B; NLRP3, NOD‐like receptor protein 3; Nrf2, nuclear factor erythroid 2‐related factor 2; VDR, vitamin D receptor.

High levels of glycolysis were observed in the lungs of acute lung injury (ALI) patients and LPS‐induced mouse models. 2‐DG pretreatment resulted in suppressed levels of glycolysis in the lung tissue of LPS‐induced ALI mice, significant improvements in histopathology, pulmonary vascular permeability, inflammatory cell infiltration, oxidative stress damage, and overexpression of pro‐inflammatory factors, and a significant increase in the survival rate of LPS‐treated mice.^24^ Lactate is an immunosuppressive factor, and high serum lactate levels are a strong negative prognostic factor in patients with severe sepsis.^59^ Lactate not only inhibits autophagy, but also reverses the promotion of autophagy by 2‐DG in cells. The protective effect on acute kidney injury (AKI) after inhibition of aerobic glycolysis is being associated with the regulation of enhanced autophagy.^44^

Lonidamine (LND) is an HK2 inhibitor whose anti‐inflammatory effects have been demonstrated in mouse models of arthritis and LPS‐induced sepsis. LND inhibits the release of tumor necrosis factorF‐α and IL‐1β caused by LPS stimulation, as well as its induced production of IL‐6 and IL‐8.^45^, ^46^

In addition to the ability of these specific glycolytic enzyme inhibitors to inhibit the inflammatory effects of sepsis, some drugs that have been shown to have anti‐inflammatory effects in animal studies also exert their anti‐inflammatory effects by modulating glycolysis.

Celastrol (Cel) is a natural anti‐inflammatory compound and the therapeutic effect of this drug on sepsis has been demonstrated in mouse models. Cel improves survival in septic mice by inhibiting the level of pro‐inflammatory cytokines. This therapeutic effect is due to the fact that Cel specifically recognizes and binds to Cys residues in PKM2 and LDHA and inhibits their activity in a dose‐dependent manner. Also Cel directly impairs the ability of HMGB1 to induce cytokine secretion.^56^

4‐Octyl itaconatek (4‐OI) alkylates Cys 22 of the glyceraldehyde 3‐phosphate dehydrogenase (GAPDH) and decreases GAPDH activity in a time‐ and dose‐dependent manner. In a mouse model of LPS‐induced sepsis, 4‐OI prevented macrophage activation and reduced serum levels of IL‐1β, IL‐6, and lactate.^57^

Tiliroside is a flavonoid with proven anti‐inflammatory effects in vivo and in vitro. It inhibits a wide range of enzymes in the glycolytic pathway including glucose transporter 1 (Glut1), enolase 1 (Eno1), and pyruvate kinase M (Pkm). PDH kinase 1 (Pdk1), Aldolase, LDHA, phosphoglycerate mutase (Pgam), phosphofructokinase (Pfk), and GAPDH, reducing the production of lactate. The inhibition of glycolysis by tiliroside modulates intestinal macrophage function and ameliorates ulcerative colitis.^58^

## 9 CONCLUSION AND PROSPECT

With the improvement of medical treatment and scientific research, the therapy and prognosis of sepsis have become more standardized. Globally, the prevalence and mortality of sepsis have improved significantly. Unfortunately, however, there are still no specific diagnostic markers or therapeutic tools for sepsis.

Early studies have successively demonstrated that high lactate levels predict a poor prognosis for patients with sepsis, the idea that sepsis is associated with reduced morbidity and mortality after correction of high lactate during hyperinflammation is well established, and early monitoring and reduction of serum lactate has been included in the latest international guidelines for the management of sepsis and septic shock. These ideas suggest that lactate somehow mediates immune disorders (especially immune paralysis) in sepsis patients, so some investigators have suggested that lactate is an immunosuppressive molecule. The discovery of lactylation modifications apparently explains at the epigenetic level the underlying cause of lactate regulation of immune status in sepsis.

While the use of lactate levels as an indicator of sepsis severity or prognosis has been consistently controversial over the past decades, the emergence of lactylation offers new hope for the diagnosis and prognosis of sepsis. Lactylation is commonly found in humans, animals, plants, microorganisms, and bacteria. Lactylation is directly regulated by lactate and is highly synergistic with lactate productio, and all factors that regulate glycolysis and affect lactate production can regulate the level of lactylation. The relative stability of lactylation as an “outcome” over time, independent of environmental changes, is one of the most important properties of lactylation as a clinical assessment indicator. Most importantly, lactylation has a bidirectional “promoting” and “inhibiting” effect compared to lactate. Under sepsis conditions, glycolysis is over‐activated, producing and accumulating large amounts of lactate, and glycolytic pathway‐related metabolic enzymes are modified by lactylation to inhibit excessive glycolytic reactions, ensuring the metabolic homeostasis of the body and limiting the level of lactate and lactylation, which is an important mechanism for maintaining the immune balance of the body. However, this feedback loop also indicates that the role of lactylation is multidimensional, and the discovery of it is only the first step in the research, and there is still a long way to go for the study of its mechanism.

Currently, the study of lactylation in sepsis is still in infancy, but it has shown a strong potential as a deeper link between lactate and immunity. Studies in the field of oncology, lactylation is extensively involved in the alteration of intrinsic and adaptive immunity, suggesting the immune status in the tumor microenvironment. Immunomodulation is also an important aspect in sepsis, and perhaps lactylation can replace lactate as one of the key molecule for more scientific and effective diagnosis and therapy.

## AUTHOR CONTRIBUTIONS

ZeXian Sun and Xin Wang, the major contributors to the manuscript, were responsible for the original draft and revision. Yu Song and Yize Li collected data, constructed the figures and tables. YongHao Yu and Xin Wang supervised the work and gave suggestions for the writing of the manuscript. All authors contributed to the article and approved the submitted version. 

## CONFLICT OF INTEREST STATEMENT

The authors declare no conflict of interest.
